# Structural Modulation and Binding of HLA-DQ8 by Cysteine-to-Serine Mutated Insulin Peptide: Insights from Molecular Dynamics Simulations

**DOI:** 10.3390/ijms27114846

**Published:** 2026-05-27

**Authors:** Rahul Mittal, Ukesh Karki, Joana R. N. Lemos, Prem Chapagain, Khemraj Hirani

**Affiliations:** 1 Diabetes Research Institute, University of Miami Miller School of Medicine, 1450 NW 10th Avenue, Miami, FL 33136, USA; 2 Division of Endocrinology, Diabetes, and Metabolism, Department of Medicine, University of Miami Miller School of Medicine, Miami, FL 33136, USA; 3 Department of Physics, Florida International University, Miami, FL 33199, USA; ukark001@fiu.edu (U.K.);; 4 Biomolecular Sciences Institute, Florida International University, Miami, FL 33199, USA

**Keywords:** Type 1 diabetes, HLA DQ8, insulin neoepitope, antigen presentation, autoimmunity, molecular dynamics simulation, peptide MHC class II interactions, binding free energy

## Abstract

Type 1 diabetes (T1D) is driven by autoreactive CD4^+^ T-cell responses to pancreatic beta cell antigens presented by disease-associated human leucocyte antigen (HLA) class II molecules. However, the molecular mechanisms by which subtle antigenic modifications promote pathogenic immunity remain incompletely defined. Recent immunopeptidomic studies have identified a cysteine-to-serine substitution at position 19 of the insulin B chain, referred to as InsC19S, as a microenvironment-driven neoepitope that can be presented by HLA class II molecules, including HLA-DQ8, and is recognized by diabetogenic CD4^+^ T cells. In this study we explore potential structural and thermodynamic mechanisms that may contribute to the enhanced immunogenicity associated with this single-amino-acid modification. Using molecular dynamics simulations combined with coarse-grained free-energy-perturbation analyses, we compared HLA DQ8 complexes bound to wild-type (WT) insulin and InsC19S peptides. The InsC19S variant is predicted in simulations to exhibit enhanced binding stability, characterized by increased hydrogen bond occupancy, reduced peptide conformational mobility, and a more favorable binding free energy. In addition, the modified peptide is predicted to induce peptide-dependent conformational adjustments within the HLA-DQ8 peptide-binding groove, resulting in expansion of the conformational landscape and stabilization of distinct low-energy states that are not accessed by the WT complex. Principal component analysis and free-energy landscape mapping suggest that this mutation may promote altered collective motions within HLA DQ8 that are consistent with enhanced peptide major histocompatibility complex (MHC) persistence and optimized antigen presentation geometry. Together, these computational observations suggest a structural framework that may help explain the preferential presentation and pathogenic recognition of InsC19S reported in experimental studies. These findings provide a molecular-level framework that may help link microenvironment-driven insulin neoepitope formation to altered peptide–MHC stability and conformational dynamics in HLA-DQ8.

## 1. Introduction

Type 1 diabetes (T1D) is an autoimmune disorder characterized by immune-mediated destruction of insulin-producing pancreatic β cells [1,2,3,4,5,6]. Autoreactive T cells play an important role in T1D pathogenesis, with both CD4^+^ and CD8^+^ T-cell populations contributing to disease development [7,8,9,10]. While CD4^+^ T cells help coordinate autoimmune responses through recognition of β-cell antigens presented by HLA class II molecules, CD8^+^ T cells can recognize β-cell-derived peptides presented by HLA class I molecules and may contribute to β-cell damage [11,12,13,14]. The onset and progression of this disease are strongly influenced by genetic predispositions, among which certain human leukocyte antigen (HLA) class II alleles, notably HLA-DQ8, play a pivotal role [15,16,17,18,19]. These molecules are integral to antigen presentation to CD4^+^ T cells and are widely recognized as one of the central determinants of susceptibility to autoimmune conditions, including T1D [20,21,22]. In individuals expressing HLA-DQ8, insulin-derived peptides are frequently presented in a manner that elicits autoreactive T-cell responses, implicating altered or context-dependent antigen presentation [11,20,23].

Recent advances in immunopeptidomics and neoantigen biology have shifted attention from conventional autoantigens to microenvironment-driven variants of self-proteins that can escape central tolerance [24,25,26]. These modifications may arise stochastically or be induced under cellular stress conditions such as inflammation, oxidative stress, or metabolic dysfunction, all of which are prevalent within pancreatic islets during the preclinical and early symptomatic stages of T1D. One such microenvironment-driven single-amino-acid variant (SAAV), a cysteine-to-serine (C-to-S) substitution in insulin, has emerged as a candidate neoepitope capable of eliciting autoreactive CD4^+^ T-cell responses in individuals with T1D [27]. This substitution, occurring at cysteine residue 19 of the insulin B chain, generates a neoepitope reported in recent immunopeptidomic and functional studies to be presented by HLA-DQ8 and recognized by diabetogenic CD4^+^ T cells [27].

Recent experimental studies in human T1D samples and the NOD mouse model have shown that this C19S-modified insulin peptide is not only processed and presented by antigen-presenting cells but also induces expansion of CD4^+^ T cells with memory-like phenotypes that persist during disease progression [27]. Importantly, this neoepitope arises from stress-induced cysteine-to-serine substitution under conditions of endoplasmic reticulum stress, oxidative redox imbalance, and proinflammatory cytokine signaling, conditions that are known to be exacerbated during insulitis. The detection of C19S insulin in both human and murine MHC class II peptidomes and its functional validation through tetramer staining, antigen presentation assays, and T-cell activation analyses together support its relevance in both disease initiation and progression [27]. Furthermore, expansion of C19S-specific HLA-DQ8-restricted CD4^+^ T cells has been observed at diabetes onset and persists into established disease, where these cells preferentially adopt an activated central memory phenotype rather than regulatory fates, sustaining long-term autoreactivity.

Despite growing evidence implicating C19S insulin as a potent neoantigen, the structural and energetic basis for its enhanced immunogenicity remains inadequately understood. While previous studies have focused on immunological recognition, there is limited insight into how this specific amino acid substitution alters peptide–MHC interactions at the atomic level, affects the stability and conformational dynamics of the MHC–peptide complex, and potentially modulates the structural plasticity of HLA-DQ8 itself. To begin addressing these gaps, we employed computational modeling approaches designed to generate structural hypotheses regarding how the C19S modification may influence peptide–MHC interactions. Specifically, we integrated atomistic molecular dynamics (MD) simulations with coarse-grained free-energy-perturbation (FEP) analysis. Using high-resolution structural models of HLA-DQ8 complexed with either the wild-type insulin peptide (InsWT) or the C19S-modified variant (InsC19S), we conducted simulations to compare binding stability, hydrogen bond formation, conformational fluctuations, and thermodynamic favorability. Furthermore, we performed free-energy landscape mapping and principal component analyses to characterize conformational transitions and dominant molecular motions induced by the peptide modification. Our simulations suggest that the C19S substitution may enhance peptide-binding stability within the HLA-DQ8 groove by increasing hydrogen bond occupancy and decreasing fluctuations in the peptide-binding groove. The mutation also induces distinct structural rearrangements in the HLA-DQ8 molecule, facilitating the formation of alternative, energetically favorable conformational states. These observations offer a possible structural interpretation that may help explain the preferential recognition of InsC19S by pathogenic T cells and support the hypothesis that even subtle peptide sequence variants can profoundly affect antigen presentation and immune recognition.

This study provides a computational structural characterization of an insulin neoepitope implicated in T1D. By elucidating how the C19S modification alters molecular interactions within the HLA-DQ8–peptide complex, we advance understanding of how neoantigen formation may contribute to autoimmune activation. Importantly, these findings are intended to generate testable hypotheses and establish a structural framework for future investigation. This framework may guide biochemical, structural, and immunological studies aimed at elucidating how stress-induced insulin neoepitopes influence HLA-DQ8 antigen presentation and subsequent T-cell recognition. Moreover, these insights may help identify structural determinants of immunogenicity and inform the rational design of peptide-based immunotherapies aimed at inducing tolerance or selectively modulating pathogenic T-cell responses in T1D.

## 2. Results

### 2.1. Cysteine-to-Serine Substitution Enhances Stability of Insulin Peptide Binding to HLA-DQ8

**Figure 1:**
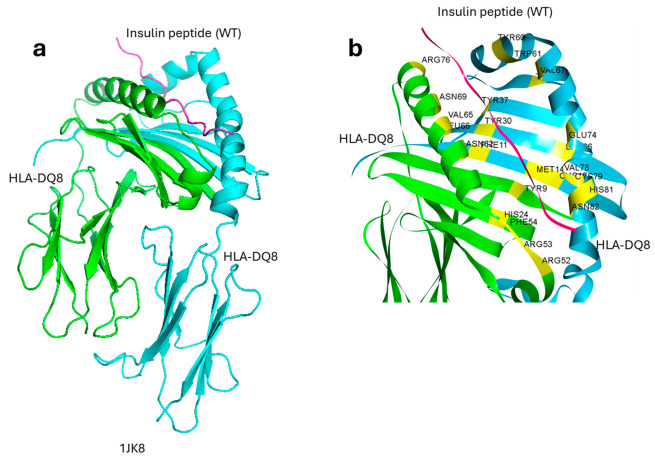
Structural model of the HLA-DQ8–insulin peptide complex. (**a**) Crystal structure of HLA-DQ8 in complex with the wild-type insulin peptide (InsWT), retrieved from the Protein Data Bank (PDB ID: 1JK8). The HLA-DQ8 α- and β-chains and the bound insulin peptide are shown in distinct colors. (**b**) Magnified protein–peptide interaction view of the HLA-DQ8 peptide-binding groove region highlighted in panel (**a**). The panel displays only the selected local interface to enhance visualization of the peptide–MHC contact region. Interacting HLA-DQ8 residues surrounding the InsWT peptide are shown to define the principal peptide-anchoring interface within the binding groove.

MD simulations were conducted to characterize the structural behavior of HLA-DQ8 in complex with either the InsWT or InsC19S (Figure 1) (Supplementary Videos S1 and S2). In both systems, the peptide remained associated with the HLA-DQ8 peptide-binding groove throughout the simulation. However, pronounced differences in conformational stability were observed between the two complexes. The InsWT peptide displayed increased conformational mobility, particularly toward the C-terminal region, with intermittent deviations relative to the peptide-binding cleft (Supplementary Figure S1). In contrast, the InsC19S peptide maintained a stable and well-defined binding pose across its entire length, exhibiting minimal positional fluctuation within the MHC class II groove (Supplementary Figure S2). These observations suggest that the cysteine-to-serine transformation may promote greater structural retention of the insulin-derived peptide within HLA-DQ8.

Analysis of representative trajectory snapshots further suggested sustained engagement of the InsC19S peptide with the peptide-binding cleft, whereas the InsWT peptide exhibited progressive flexibility and partial disengagement along the binding interface (Supplementary Figures S1 and S2). Collectively, these results suggest that substitution at position 19 may stabilize peptide–MHC interactions and restrict conformational freedom within the complex.

### 2.2. Increased Hydrogen Bond Occupancy Stabilizes the InsC19S–HLA-DQ8 Complex

**Figure 2:**
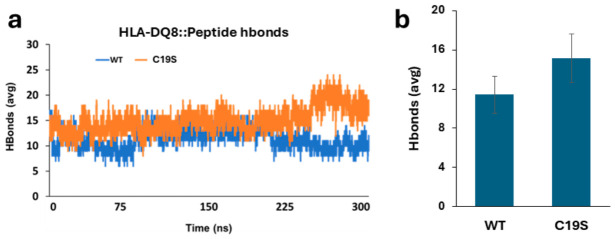
Hydrogen bond analysis of insulin peptide–HLA-DQ8 complexes. (**a**) Time-resolved number of intermolecular hydrogen bonds formed between HLA-DQ8 and InsWT or InsC19S peptides over the full 300 ns molecular dynamics simulations. (**b**) Average hydrogen bond occupancy between each peptide variant and HLA-DQ8 calculated across the 300 ns trajectories. The InsC19S peptide consistently forms a greater number of hydrogen bonds, indicating enhanced interaction stability within the MHC class II binding groove. Error bars indicate the standard deviation (SD) derived from all sampled frames throughout the simulation trajectory.

Quantitative assessment of intermolecular hydrogen bonding revealed an increase in hydrogen bond occupancy in the InsC19S–HLA-DQ8 complex relative to the InsWT complex (Figure 2a). Across the simulation, the InsC19S peptide consistently formed a higher number of hydrogen bonds with residues lining the HLA-DQ8 peptide-binding groove. The average hydrogen bond count for the InsC19S complex exceeded that of the InsWT complex by approximately 35% (Figure 2b), reflecting a more extensive and persistent interaction network. This enhanced hydrogen bonding was associated in simulations with reduced structural fluctuations of both the peptide and the surrounding MHC residues. Together, these observations suggest a more stable peptide–MHC interaction interface.

### 2.3. Thermodynamic Analysis Suggests Enhanced Binding Affinity of InsC19S for HLA-DQ8

**Figure 3:**
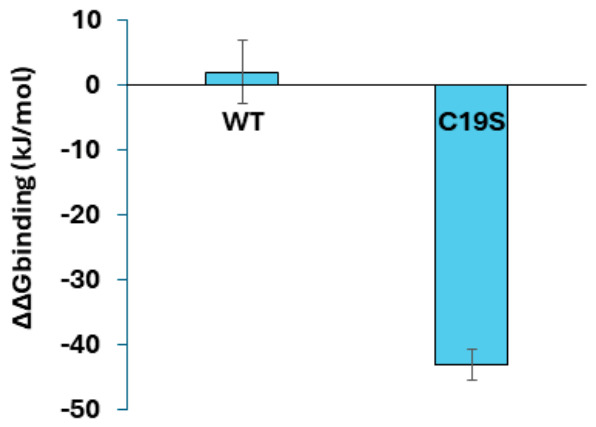
**Binding-free-energy differences between insulin peptide variants.** Relative binding-free-energy differences (ΔΔG_binding) for InsWT and InsC19S peptides in complex with HLA-DQ8, calculated using coarse-grained free-energy-perturbation simulations. ΔΔG_binding values were obtained using the thermodynamic cycle ΔΔG_binding = ΔG_AB − (ΔG_A + ΔG_B), where AB represents the peptide–HLA-DQ8 complex and A and B represent the unbound protein and peptide, respectively. The InsC19S peptide displays a markedly more favorable binding free energy than the wild-type peptide. Data are presented as mean ± SEM, where error bars indicate the standard error of the mean.

To evaluate the energetic consequences of the cysteine-to-serine substitution, binding free energies were calculated using coarse-grained free-energy-perturbation simulations. The InsC19S peptide exhibited a strongly favorable binding free energy in complex with HLA-DQ8, whereas the InsWT peptide displayed a weak and near-neutral binding profile (Figure 3). The magnitude of the binding-free-energy difference is consistent with the possibility that substitution at position 19 may enhance the thermodynamic favorability of peptide binding to HLA-DQ8. This thermodynamic stabilization provides a quantitative basis for the increased persistence of the InsC19S peptide within the MHC class II groove.

### 2.4. Mutated Peptide Induces Altered Conformational Dynamics of HLA-DQ8

**Figure 4:**
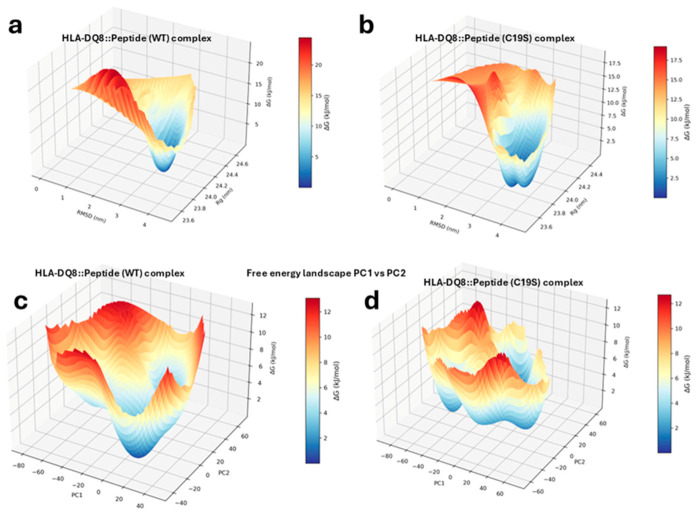
Free-energy landscapes derived from 300 ns molecular dynamics simulations. Free-energy landscapes constructed from 300 ns simulation trajectories using backbone root mean square deviation (RMSD) and radius of gyration (Rg) as collective variables. (**a**,**c**) Energy landscapes for the HLA-DQ8–InsWT complex. (**b**,**d**) Energy landscapes for the HLA-DQ8–InsC19S complex. Free-energy values were calculated from conformational probabilities using Boltzmann inversion. The InsC19S-bound complex samples a broader distribution of low-energy conformations, indicating increased conformational heterogeneity relative to InsWT.

To determine whether enhanced peptide binding influenced the structural behavior of the HLA-DQ8 molecule, free-energy landscapes were constructed using root mean square deviation and radius of gyration as collective variables. The InsWT–HLA-DQ8 complex occupied a single, narrow energy minimum, consistent with a relatively constrained conformational ensemble (Figure 4a,c). In contrast, the InsC19S–HLA-DQ8 complex populated a broader and more heterogeneous energy landscape, consistent with increased conformational plasticity (Figure 4b,d).

**Figure 5:**
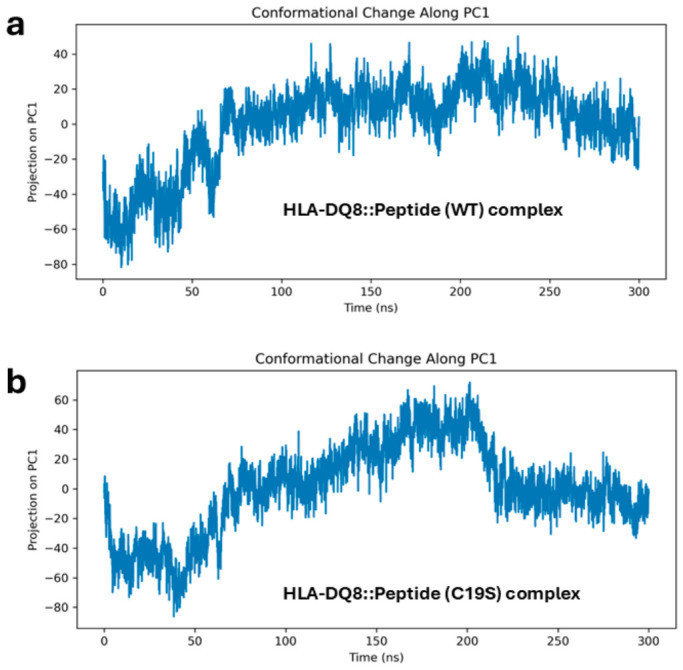
Principal component analysis of peptide-dependent conformational dynamics. Projection of molecular dynamics trajectories onto the first two principal components (PC1 and PC2) derived from backbone atomic fluctuations of the peptide–HLA-DQ8 complexes. (**a**) The InsWT complex is characterized by a single dominant conformational basin, whereas (**b**) the InsC19S complex accesses multiple distinct low-energy basins, reflecting altered collective motions induced by the peptide modification.

Principal component analysis of the simulation trajectories suggested distinct dynamic signatures between the two complexes. The InsWT complex was characterized by a single dominant conformational basin, whereas the InsC19S complex accessed multiple energetically favorable basins separated by defined barriers (Figure 4c,d). Projection of the trajectories along the principal components suggested a stabilized binding mode unique to the InsC19S–HLA-DQ8 complex (Figure 5).

### 2.5. Structural Remodeling of the Peptide-Binding Groove in the InsC19S Complex

**Figure 6:**
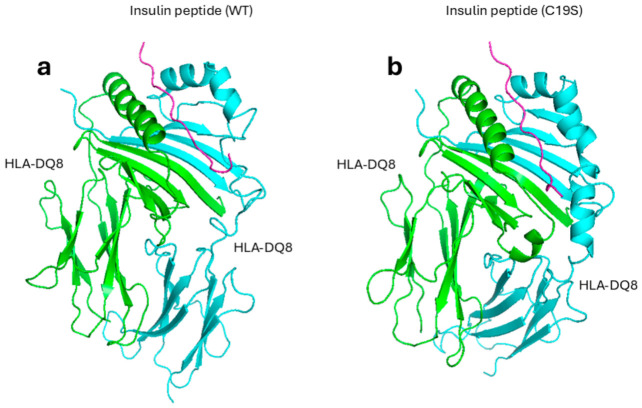
Structural remodeling of the HLA-DQ8 peptide-binding groove. Representative final structures extracted at 300 ns from molecular dynamics simulations. (**a**) HLA-DQ8 bound to InsWT, showing a canonical peptide-binding groove with increased peptide flexibility. (**b**) HLA-DQ8 bound to InsC19S, demonstrating deeper peptide embedding and localized structural rearrangements within the peptide-binding groove. Structures are colored by chains to emphasize peptide positioning and MHC architecture.

Structural analysis revealed that the HLA-DQ8 complex bound to the InsWT retained a canonical peptide-binding groove architecture and exhibited greater peptide flexibility (Figure 6a). In contrast, binding of the InsC19S induced subtle yet reproducible rearrangements within the HLA-DQ8 peptide-binding groove. The substituted peptide adopted a more deeply embedded and conformationally restrained orientation within the binding cleft, accompanied by localized adjustments in the surrounding α-helical and β-sheet elements that define the MHC class II groove (Figure 6b).

Together, these results suggest that a single cysteine-to-serine substitution at position 19 of an insulin-derived peptide may substantially enhance peptide–MHC binding stability, strengthen intermolecular interactions, reshape the thermodynamic landscape, and induce conformational remodeling of the HLA-DQ8 molecule.

## 3. Discussion

T1D is characterized by a progressive failure of immune tolerance to insulin-producing β cells, driven by autoreactive T cells recognizing β-cell-derived antigens presented by disease-associated HLA class II molecules [1,2]. Although insulin has been recognized as a primary autoantigen in T1D, the molecular determinants that enable specific insulin epitopes to persistently engage autoreactive CD4^+^ T cells remain incompletely defined [15,28]. In this study, we provide a structural and energetic framework that suggests that a single cysteine-to-serine substitution at position 19 of an insulin-derived peptide may alter its interaction with HLA-DQ8, potentially yielding a peptide–MHC with enhanced stability, altered conformational dynamics, and features that may be consistent with enhanced autoimmune relevance [29,30].

Our findings are consistent with recent immunopeptidomic and functional data showing that the InsC19S variant represents a verified microenvironment-driven insulin neoepitope generated in stressed pancreatic islets and cytokine-activated antigen-presenting cells. This neoepitope is presented by T1D-predisposing HLA class II molecules, including HLA-DQ8 [31]. This study further supports the characterization of C19S as a microenvironment-driven, single-amino-acid neoantigen that elicits expansion of register-specific CD4^+^ T cells with memory-like properties that persist throughout disease progression. The molecular mechanisms underlying the immunological dominance of this neoepitope, however, were not previously defined. Our results begin to address this gap by suggesting that the C19S substitution may enhance peptide–HLA-DQ8 binding stability and alter the conformational landscape of the complex. Our findings are in agreement with previous studies indicating that peptide chemistry may influence MHC-II dynamics and stability [23,32,33].

Although recent experimental studies have shown the presence and immunological relevance of the C19S insulin neoepitope in T1D, the structural mechanisms governing its interaction with disease-associated HLA molecules remain incompletely defined. By integrating molecular dynamics simulations with free-energy-perturbation analyses, the present study provides a complementary structural framework to examine how this single-residue modification may influence peptide–MHC stability and conformational dynamics. These computational insights extend prior experimental observations by proposing potential molecular mechanisms through which the C19S substitution could modulate antigen presentation and subsequent T-cell recognition in the context of HLA-DQ8.

A central implication of our work is that minimal peptide modifications may influence antigen presentation by stabilizing peptide engagement within disease-associated MHC-II molecules [34,35]. Compared with the InsWT, the InsC19S variant exhibits reduced conformational mobility, deeper positioning within the HLA-DQ8 binding cleft, and a denser intermolecular hydrogen-bonding network. These properties are accompanied by a marked increase in binding free energy, indicating that the substituted peptide occupies a more energetically favorable state. In the context of T1D, increased peptide–MHC stability may be relevant, as it could contribute to differences in the persistence and surface density of peptide–MHC complexes on antigen-presenting cells, which may influence the likelihood of productive encounters with autoreactive T cells [29,30,36,37].

Importantly, our data indicates that the impact of the C19S substitution extends beyond peptide affinity to encompass peptide-induced remodeling of the HLA-DQ8 molecule itself. Free-energy landscape and PCA reveal that complexes containing the C19S peptide populate a broader ensemble of low-energy conformational states than those bound to the WT peptide. The free-energy landscape analysis further revealed that the HLA-DQ8–InsC19S complex samples two distinct low-energy minima, whereas the HLA-DQ8–InsWT complex predominantly occupies a single major energy basin. This observation suggests that the C19S substitution may increase conformational heterogeneity and flexibility within the peptide-bound complex, potentially enabling transitions between multiple metastable states. In contrast, the WT complex appears to maintain a comparatively more restricted conformational ensemble. Although the present study was not specifically designed to characterize transition pathways between these states, the observed dual-minima behavior in the InsC19S system may reflect altered peptide accommodation dynamics within the HLA-DQ8 binding groove. Further long-timescale simulations and cluster-based structural analyses would help define the representative conformations associated with these minima and their potential biological relevance. Taken together, these findings suggest that C19S-associated changes in peptide accommodation may contribute to enhanced conformational plasticity of the HLA-DQ8 peptide-binding groove. This enhanced conformational plasticity suggests that the peptide-binding groove of HLA-DQ8 is dynamically responsive to subtle changes in peptide chemistry. Such peptide-dependent modulation of MHC-II dynamics is increasingly recognized as a determinant of T-cell receptor (TCR) engagement, as it can influence the geometry, stability, and lifetime of the peptide–MHC surface presented for immune surveillance [38,39].

Our computational analyses provide a structural hypothesis that may help explain the enhanced immunogenicity of the C19S insulin neoepitope reported in recent experimental studies. The results of this study suggest that C19S-specific CD4^+^ T cells preferentially recognize defined peptide registers and exhibit transcriptional programs associated with activation, clonal expansion, and central memory differentiation. Our observation that the InsC19S peptide adopts a more deeply embedded and conformationally restrained orientation within the HLA-DQ8 groove suggests a presentation state that may preferentially stabilize particular registers while reducing conformational heterogeneity. Such stabilization could sharpen the molecular features sensed by TCRs, thereby favoring selective expansion of C19S-reactive clones [28].

From a broader perspective, our results are consistent with an emerging paradigm in autoimmune diabetes in which neoepitope formation does not require large peptide modifications or fusion events. Instead, context-dependent SAAV arising from β-cell stress and inflammation can be sufficient to generate structurally and energetically distinct peptide–MHC complexes [24,40]. In the case of C19S, oxidative remodeling of insulin within stressed islets and antigen-presenting cells generates a peptide variant that is immunogenic and structurally favorable for presentation by HLA-DQ8. This convergence of microenvironment-driven peptide modification and MHC-II structural permissiveness offers a possible explanation for the sustained autoreactivity observed in T1D.

In interpreting these findings, it is important to note that the present simulations were designed to evaluate the structural consequences of the C19S substitution after its formation, rather than to model the oxidative process that generates it. Accordingly, the simulations were performed under standard classical MD conditions and did not explicitly incorporate oxidative stress, redox reactions, or additional oxidative post-translational modifications. Therefore, the C19S variant was evaluated primarily in terms of peptide–HLA-DQ8 stability, intermolecular interactions, and conformational dynamics. Nevertheless, as cysteine residues are chemically sensitive to oxidative modification, replacement of cysteine with serine may represent a structural consequence of stress-associated remodeling within the islet microenvironment. In this context, the altered hydrogen-bonding pattern, reduced peptide mobility, and changes in the HLA-DQ8 conformational landscape observed for the InsC19S complex may provide indirect structural insight into how microenvironment-associated peptide remodeling could influence antigen presentation. Future simulations incorporating redox-sensitive parameters, alternative cysteine oxidation states, or stress-associated peptide modifications may help elucidate the potential influence of oxidative conditions on insulin neoepitope presentation in T1D.

Our findings also align with previous studies suggesting that T1D-associated HLA-DQ molecules may exhibit distinctive peptide-binding properties and altered susceptibility to peptide editing, which may amplify the impact of intrinsically stable autoantigenic peptides. In such a permissive presentation environment, even modest increases in peptide–MHC stability may translate into disproportionately large effects on immune recognition. The C19S substitution may represent an example of this principle, potentially giving rise to a structurally reinforced insulin epitope that could be associated with chronic autoimmune responses.

There are limitations of the present study that should be considered. First, the conclusions are derived from computational simulations and energetic analyses, which provide high-resolution insight into peptide–MHC interactions. The difference in hydrogen bond counts between the InsWT and InsC19S complexes became more apparent during the later portion of the trajectory, particularly after approximately 225 ns. Although this trend was maintained through the remaining simulation period, its late emergence should be interpreted with appropriate caution. Other stability metrics, including RMSD, radius of gyration, and overall conformational behavior, did not indicate major global instability during this interval. Nevertheless, extended simulations would provide additional sampling and may help determine whether the observed late-stage hydrogen-bonding differences remain stable over longer timescales. This consideration is consistent with the broader limitations of computational simulations, which cannot fully capture the complexity of antigen processing, intracellular trafficking, and surface presentation in living cells. The present interaction analysis focused primarily on intermolecular hydrogen bonding as a readily quantifiable measure of peptide–HLA-DQ8 interaction stability across the simulation trajectories. Other nonbonded interaction components, including hydrophobic contacts, van der Waals interactions, electrostatic contacts, and residue-level contact networks, were not systematically decomposed in the current analysis. As the C19S substitution replaces a sulfur-containing side chain with a polar hydroxyl-containing residue, it may influence local packing, side-chain accommodation, and nonbonded interaction patterns within the peptide-binding groove. More detailed residue-level contact mapping or energy decomposition analyses would therefore be useful in future studies to further define the contribution of non-hydrogen-bond interactions to the altered behavior of the InsC19S–HLA-DQ8 complex. Second, the analysis focuses on a single insulin-derived peptide and a single MHC class II allele, HLA-DQ8, and therefore does not capture the full diversity of insulin epitopes or HLA class II molecules implicated in T1D. Third, although enhanced peptide–MHC stability and conformational remodeling are consistent with increased immunological relevance, direct effects on TCR binding kinetics and downstream T-cell activation were not explicitly modeled. In addition, the simulations do not account for the influence of peptide flanking residues, HLA-DM-mediated peptide editing, or the cellular microenvironment, all of which can modulate antigen presentation in vivo. Finally, while the structural differences observed are robust across multiple analyses, experimental validation will be required to directly link these molecular features to functional immune outcomes. Interestingly, some experimental systems have suggested that C19S peptides may exhibit altered MHC binding characteristics depending on the presenting molecule and register context. Our simulations specifically examine the HLA-DQ8 complex and should therefore be interpreted as a structural model for one potential presentation pathway rather than a universal explanation across all MHC class II contexts.

Despite these limitations, our findings indicate that microenvironment-driven single-residue modification of self-peptide may influence MHC class II behavior at the molecular level. The cysteine-to-serine substitution within the insulin epitope generates a peptide–MHC with distinct thermodynamic stability and dynamic properties, suggesting that peptide sequence variation may allosterically influence the conformational states of HLA-DQ8 rather than merely modulating binding affinity. Future experimental studies integrating structural biology, peptide–MHC binding assays, and T-cell functional analyses will be important to directly test the hypotheses generated by these simulations. In particular, high-resolution structural determination of HLA-DQ8 bound to the C19S peptide, using X-ray crystallography or cryo-electron microscopy, would provide critical validation of the interaction networks and conformational states predicted in our simulations. This study proposes a structural framework that may contribute to understanding how the C19S insulin neoepitope could be preferentially presented by HLA-DQ8. Importantly, the computational approach established here may be extended to modeling other neoepitopes and their interactions with disease-associated MHC molecules. Such applications could provide a generalizable strategy for investigating the structural determinants of neoepitope presentation and advancing mechanistic understanding of autoimmune pathogenesis. These findings highlight the utility of computational approaches for generating experimentally testable hypotheses regarding neoepitope-driven autoimmunity in T1D. Importantly, this framework may inform therapeutic strategies aimed at selectively destabilizing pathogenic peptide–MHC conformations or modulating MHC class II dynamics, which could contribute to antigen-specific regulation of autoimmune T-cell responses in T1D.

## 4. Materials and Methods

### 4.1. Structure Retrieval and In Silico Peptide Modification

The three-dimensional structure of the HLA-DQ8 molecule bound to the wild-type insulin peptide (InsWT) was retrieved from the Protein Data Bank (PDB) under accession code 1JK8 [41]. Structural preprocessing was carried out using Discovery Studio Visualizer (DS), during which crystallographic water molecules were removed, and hydrogen atoms were added according to physiological protonation states. A cysteine-to-serine substitution was introduced at position 19 of the insulin peptide to generate the C19S-modified insulin variant (InsC19S) using the mutagenesis tool implemented in DS. Following mutation, local energy minimization was performed to alleviate steric strain while preserving the peptide backbone conformation and maintaining the native architecture of the HLA-DQ8 peptide-binding groove. The resulting HLA-DQ8–InsWT and HLA-DQ8–InsC19S complexes were used as starting structures for all subsequent computational analyses.

### 4.2. Atomistic Molecular Dynamics Simulation

Atomistic MD simulations were performed using the GROMACS simulation package [42,43,44,45]. Simulations were conducted independently for the HLA-DQ8 complexed with InsWT and InsC19S using identical protocols to ensure direct comparability. Each system was placed in a triclinic simulation box and solvated with Simple Point Charge (SPC) water molecules to ensure adequate hydration [46]. System neutrality was achieved by the addition of sodium and chloride ions, followed by the inclusion of additional sodium chloride to reach a final ionic strength of 0.15 M, approximating physiological conditions. All systems were parameterized using the Optimized Potentials for Liquid Simulations (OPLS) force field [47]. Energy minimization was carried out using the steepest descent algorithm for 5000 steps to remove unfavorable contacts. The systems were subsequently equilibrated under constant volume and temperature (NVT) conditions, followed by constant pressure and temperature (NPT) equilibration for a total of 300 picoseconds. Production MD simulations were then performed for 300 nanoseconds (ns) using a leap-frog integrator, with atomic coordinates recorded every 50 picoseconds for downstream analysis. Independent simulations were conducted to verify the robustness of the observed trends. The 300 ns simulation duration was selected to provide sufficient conformational sampling and stabilization of the peptide–HLA-DQ8 complexes, considering the dynamic nature of peptide-bound MHC systems. This timescale is commonly used in MD studies of peptide–protein interactions and allows assessment of key stability parameters, including RMSD, radius of gyration, and hydrogen-bonding profiles [48,49,50]. The objective of the simulations was to evaluate the relative stability and conformational behavior of the InsWT and InsC19S complexes under matched, physiologically relevant conditions.

### 4.3. Trajectory Processing and Structural Stability Analysis

MD trajectories were processed using the built-in GROMACS analysis tools [42,43,44,45]. Structural stability of the peptide–HLA-DQ8 complexes was evaluated by calculating the backbone root mean square deviation (RMSD) relative to the initial structure over the entire 300 ns simulation period [51,52,53,54]. Residue-level flexibility was assessed using root mean square fluctuation (RMSF) analysis [55,56,57,58,59]. Global compactness of the complexes was quantified by calculating the radius of gyration (Rg) [60,61,62]. These metrics were used to compare conformational stability and dynamic behavior between the HLA-DQ8–InsWT and HLA-DQ8–InsC19S systems. Representative structural snapshots, including the final 300 ns conformations, were extracted to visualize peptide positioning and engagement within the HLA-DQ8 peptide-binding groove.

### 4.4. Intermolecular Hydrogen Bond Analysis

Intermolecular hydrogen bonds between the insulin peptides and HLA-DQ8 were quantified over the full 300 ns simulation trajectories. Hydrogen bonds were defined using standard geometric criteria based on donor–acceptor distance and angular cutoffs. The total number of peptide–HLA-DQ8 hydrogen bonds was calculated as a function of simulation time, and average hydrogen bond occupancies were determined for each complex. These analyses were used to assess the persistence and stability of peptide–MHC interactions and to identify differences in interaction networks induced by the C19S substitution.

### 4.5. Free-Energy Landscape and Principal Component Analysis

To characterize the conformational space sampled during the 300 ns MD simulations, free-energy landscapes (FELs) were constructed using backbone RMSD and Rg as collective variables. Probability distributions were converted into free-energy surfaces using Boltzmann inversion [63,64,65]. Principal component analysis (PCA) was performed on backbone atoms of the peptide–HLA-DQ8 complexes to identify dominant collective motions governing system dynamics. Trajectories were projected onto the first two principal components to enable direct comparison of conformational sampling between the InsWT- and InsC19S-bound systems. Differences in conformational basins and energy minima were interpreted as peptide-induced modulation of HLA-DQ8 dynamics.

### 4.6. Coarse-Grained Free-Energy-Perturbation Calculations

Binding-free-energy differences between InsWT and InsC19S were calculated using coarse-grained free-energy-perturbation (FEP) simulations based on the Martini coarse-grained force field [66,67,68]. Atomistic structures were converted into coarse-grained representations and placed in dodecahedral simulation boxes. Each system was briefly minimized in vacuo, solvated with coarse-grained water, and subjected to an additional 5000-step energy minimization. Free-energy calculations were performed using 11 gamma coupling parameters ranging from 0 to 10 to interpolate between initial and final states. For each gamma value, an independent 90 ns MD simulation was conducted. Three systems were simulated for each peptide variant, including the peptide–HLA-DQ8 complex (AB), the isolated HLA-DQ8 molecule (A), and the isolated peptide (B). Gibbs free energies (ΔG) were calculated for each system, and binding-free-energy differences were determined using the relationship ΔΔGbinding = ΔGAB − (ΔGA + ΔGB). The cumulative simulation time for each peptide system exceeded 3.6 microseconds to improve thermodynamic sampling.

### 4.7. Visualization

Trajectory visualization and structural renderings were generated using the DS- and GROMACS-compatible visualization tools. Hydrogen bond statistics, free-energy landscapes, PCA projections, and all quantitative plots were generated using Python-based analysis pipelines (version 3.10.12), allowing for a comprehensive assessment of the dynamic behavior and stability of the target protein–ligand complex throughout the simulation period.

## Data Availability

The original contributions presented in this study are included in the article/Supplementary Materials. Further inquiries can be directed to the corresponding authors.

## Supplementary Materials

The following supporting information can be downloaded at: https://www.mdpi.com/article/10.3390/ijms27114846/s1.
