# Supplementary material for: Structural Modulation and Binding of HLA-DQ8 by Cysteine-to-Serine Mutated Insulin Peptide: Insights from Molecular Dynamics Simulations
Source: Int J Mol Sci. 2026 May 27;27(11):4846. doi: 10.3390/ijms27114846 (PMC13256993; doi:10.3390/ijms27114846)
Supplement: Supplementary file 1 [file ijms-27-04846-s001.zip › Supplementary Videos.pptx]

## Slide 1
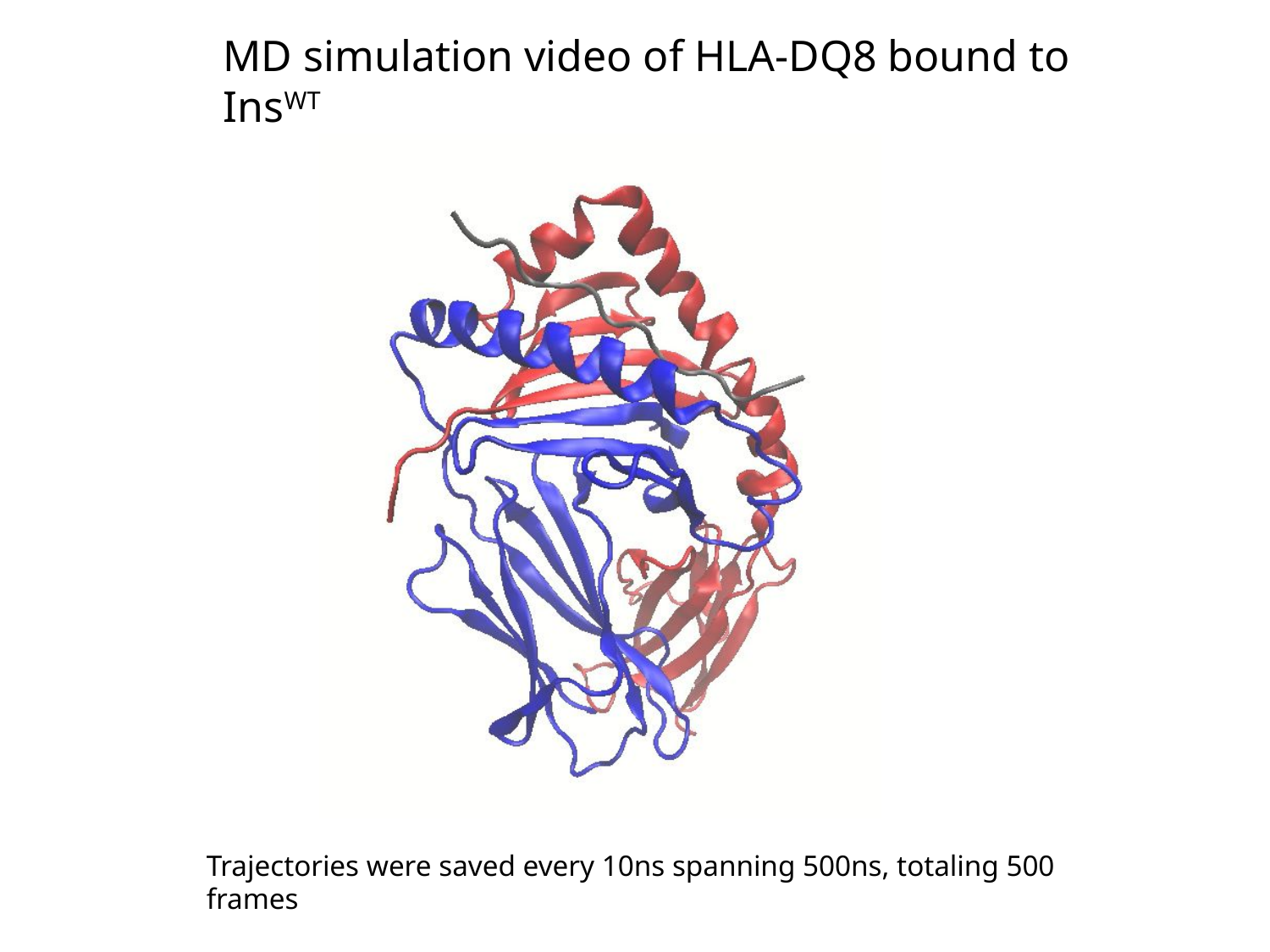

MD simulation video of HLA-DQ8 bound to InsWT
Trajectories were saved every 10ns spanning 500ns, totaling 500 frames

## Slide 2
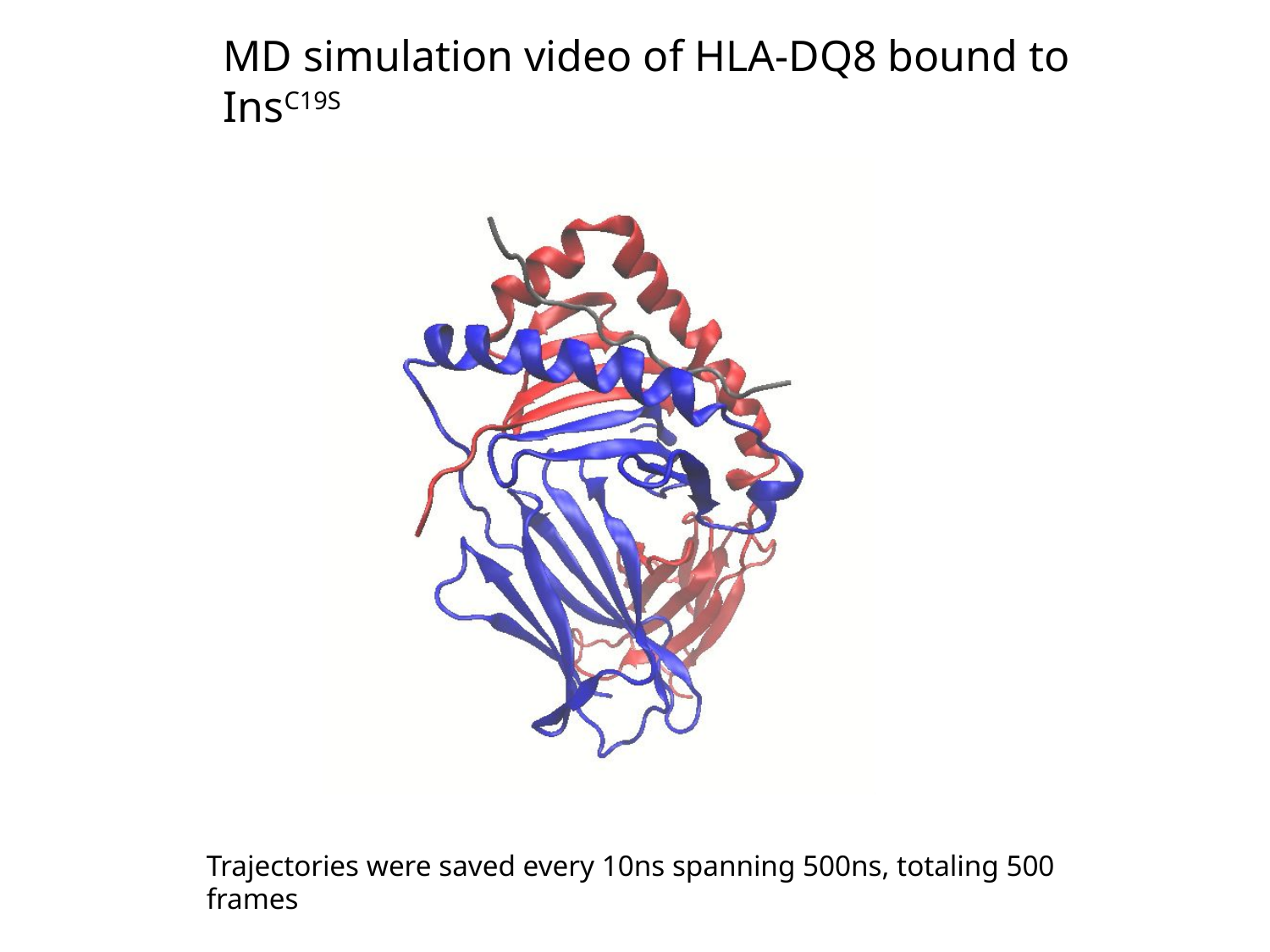

MD simulation video of HLA-DQ8 bound to InsC19S
Trajectories were saved every 10ns spanning 500ns, totaling 500 frames
