# Supplementary material for: Structural Modulation and Binding of HLA-DQ8 by Cysteine-to-Serine Mutated Insulin Peptide: Insights from Molecular Dynamics Simulations
Source: Int J Mol Sci. 2026 May 27;27(11):4846. doi: 10.3390/ijms27114846 (PMC13256993; doi:10.3390/ijms27114846)
Supplement: Supplementary file 1 [file ijms-27-04846-s001.zip › Supplementray figure Legends.pdf]

Supplementary Figure Legends

Supplementary Figure 1. Conformational evolution of the HLA-DQ8–wild-type insulin peptide complex during molecular dynamics simulation. Representative structural snapshots of the HLA-DQ8 molecule in complex with the wild-type insulin peptide (InsWT) extracted at 0, 50, 100, 150, 200, 250, and 300 ns from a 300 ns atomistic molecular dynamics simulation. The N- and C-termini of the peptide are labeled to visualize time-dependent changes in peptide positioning within the HLA-DQ8 peptide-binding groove. Although the peptide remains associated with the groove throughout the simulation, increasing conformational variability is observed, particularly toward the C-terminal region, leading to progressive rearrangement within the binding cleft. Such dynamic instability may reduce the lifetime and surface persistence of peptide–HLA complexes, thereby limiting efficient presentation to autoreactive $CD4^+$ T cells. In the context of type 1 diabetes, reduced stability of native insulin peptides may constrain their immunogenic potential under physiological conditions.

Supplementary Figure 2. Stabilized binding configuration of the C19S-modified insulin peptide within the HLA-DQ8 peptide-binding groove and implications for autoimmune activation. Structural snapshots of the HLA-DQ8 molecule bound to the C19S-modified insulin peptide (InsC19S) captured at 0, 50, 100, 150, 200, 250, and 300 ns during a 300 ns molecular dynamics simulation. The N- and C-termini of the peptide are indicated to facilitate assessment of peptide orientation and positional stability. Across all time points, the InsC19S peptide maintains a highly persistent and well-defined binding pose, exhibiting minimal conformational drift within the peptide-binding groove. Sustained peptide engagement of this nature is expected to enhance the stability and surface density of peptide–HLA-DQ8 complexes on antigen-presenting cells, thereby increasing the probability of productive encounters with insulin-reactive $CD4^+$ T cells. In type 1 diabetes, such prolonged and stable presentation of a modified insulin epitope may promote the expansion and maintenance of pathogenic T cell populations, contributing to the breakdown of immune tolerance and the persistence of autoimmune responses.
