# Supplementary figures and images for: Structural Modulation and Binding of HLA-DQ8 by Cysteine-to-Serine Mutated Insulin Peptide: Insights from Molecular Dynamics Simulations
Source: Int J Mol Sci. 2026 May 27;27(11):4846. doi: 10.3390/ijms27114846 (PMC13256993; doi:10.3390/ijms27114846)

Supplementary Figure 1

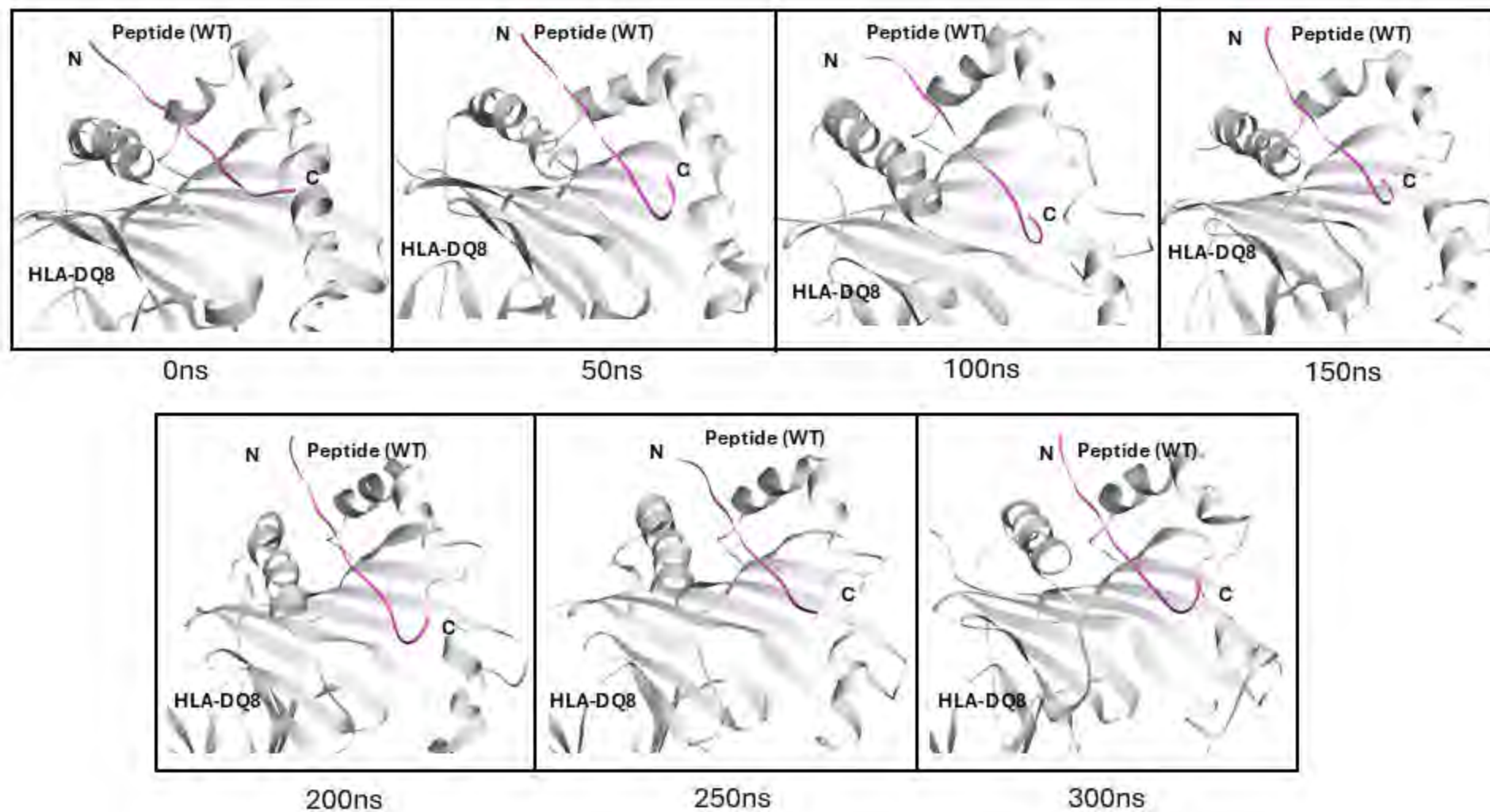

Supplement: Supplementary file 1 [file ijms-27-04846-s001.zip › Supplementary Figure S1.pdf]

Supplementary Figure 2

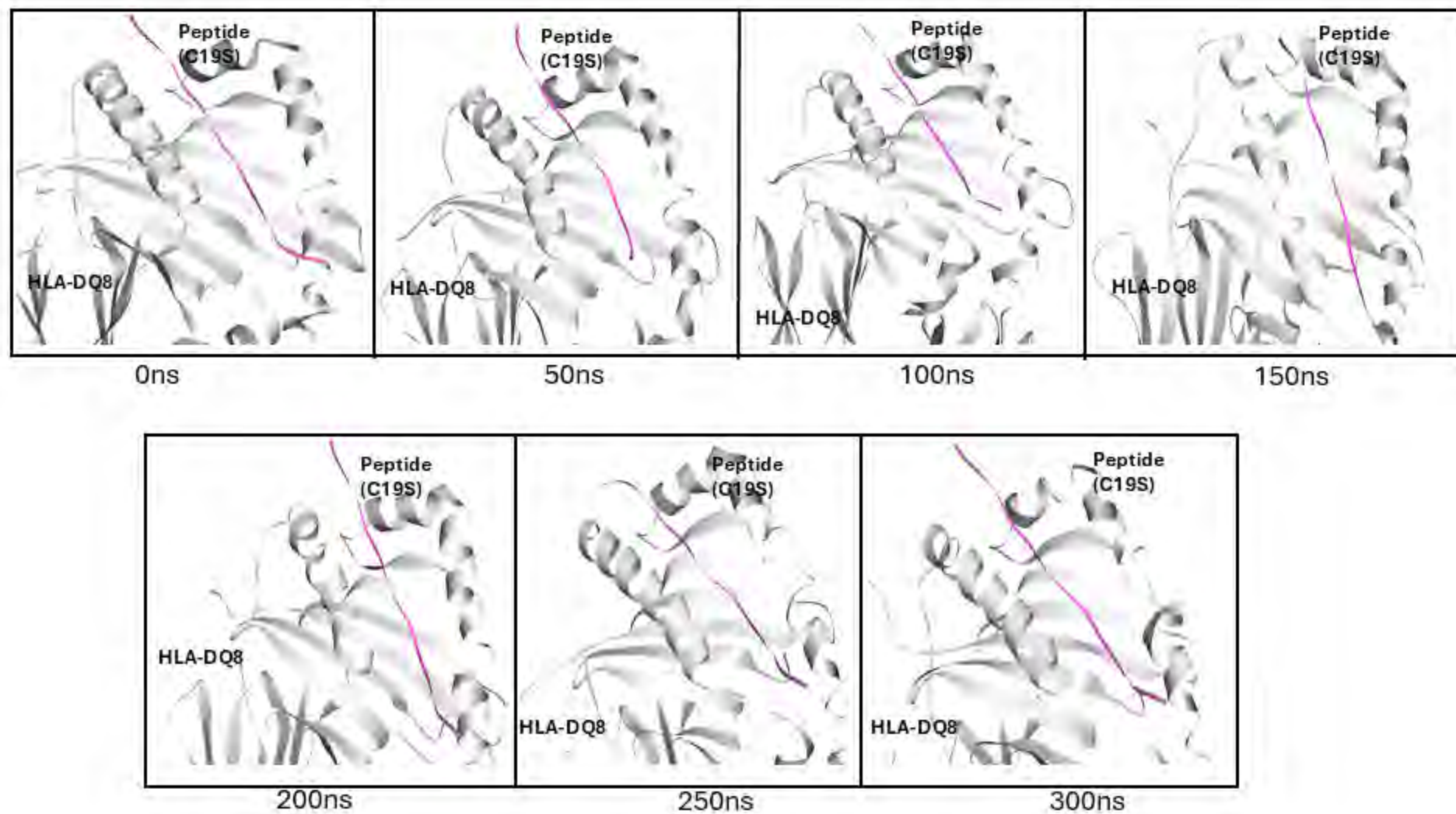

Supplement: Supplementary file 1 [file ijms-27-04846-s001.zip › Supplementary Figure S2.pdf]
